# Nomogram Prediction of Anastomotic Leakage and Determination of an Effective Surgical Strategy for Reducing Anastomotic Leakage after Laparoscopic Rectal Cancer Surgery

**DOI:** 10.1155/2017/4510561

**Published:** 2017-05-16

**Authors:** Chang Hyun Kim, Soo Young Lee, Hyeong Rok Kim, Young Jin Kim

**Affiliations:** Department of Surgery, Chonnam National University Hwasun Hospital and Medical School, Hwasun, Chonnam, Republic of Korea

## Abstract

**Background:**

Although many surgical strategies have been used to reduce the anastomotic leak (AL) rate after laparoscopic rectal cancer surgery, limited data are available on the risk factors for AL and the effective strategy to reduce AL.

**Methods:**

The present study enrolled 736 consecutive patients who underwent laparoscopic resection without a diverting stoma for rectal adenocarcinoma. A nomogram was constructed to predict AL. Based on the nomogram, personalized risk was calculated and sequential surgical strategies were monitored using risk-adjusted cumulative sum (RA-CUSUM) analysis.

**Results:**

Among the 736 patients, clinical AL occurred in 65 patients (8.8%). Sex, an American Society of Anesthesiologists score, operation time, blood transfusion, and tumor location were identified as significant predictive factors for AL. Based on these factors, a nomogram was created to predict AL, with a concordance index (C-index) of 0.753 (95% confidence interval, 0.690–0.816). A calibration plot showed good statistical performance on internal validation (bias-corrected C-index of 0.742). The RA-CUSUM curve showed that extended splenic flexure mobilization (SFM) could be the most influential strategy to reduce AL.

**Conclusions:**

Our nomogram for predicting AL after laparoscopic rectal cancer surgery might be helpful to identify the individual risk of AL. Furthermore, extended SFM might be the most appropriate strategy for reducing AL.

## 1. Introduction

Colorectal cancer (CRC) is a major cause of cancer mortality and morbidity, and it has been reported that this cancer contributes to approximately 10% of the cancer mortality rate [1]. The introduction of total mesorectal excision (TME) and preoperative chemoradiotherapy (CRT) for rectal cancer has dramatically improved the oncological outcome, especially in terms of local recurrence [2, 3]. The use of abdominoperineal resection (APR) varies widely across the world, and its use has been constantly decreasing. It is believed that TME and preoperative CRT have increased the rate of sphincter preservation in patients with mid-to-low rectal cancer [4–6]. The use of sphincter-preserving surgery has increased, and this might contribute to an increase in the incidence of anastomotic leakage (AL) [7]. AL is an important factor that can not only increase the postoperative morbidity and mortality rates but also reduce the quality of life [8, 9]. Furthermore, its influence on the oncological outcome is debatable, and some authors have suggested that AL might be associated with an increase in the local recurrence rate and a reduction in cancer-related survival [10, 11]. The incidence of AL after rectal anastomosis has been reported to vary from 3% to 21%, with higher rates after emergency surgery [12–18].

Many attempts have been made to decrease the rate of AL after rectal cancer surgery. A diverting stoma has been reported to reduce the rate of anastomotic failure; however, this remains controversial [19, 20]. In addition, a diverting stoma can cause stoma-related complications, and the additional operation for stoma closure is associated with further morbidity, mortality, and economical cost [21]. In a previous study, among patients in whom a temporary diverting stoma was planned preoperatively, approximately 20% who experienced anastomotic complications or tumor progression with local recurrence and distant metastasis did not undergo stoma closure, and the stoma was left in situ in these patients [9]. Therefore, a diverting stoma should be avoided as much as possible. Several other strategies, such as the application of fibrin glue [14], the use of reinforcing sutures [22], splenic flexure takedown [23], and the use of a transanal drain tube [24], have been adapted to decrease the incidence of AL.

Various strategies have been sequentially used at our institution to reduce the incidence of AL after laparoscopic rectal cancer surgery. A direct comparison of the strategies might result in serious selection bias and failure to obtain a high clinical significance. Therefore, the development of a prediction model of AL after surgery for rectal cancer and the determination of the risk-reducing factors in controllable strategies are very important. In this regard, the present study aimed to construct a prediction model and identify the most effective strategy for reducing AL in patients treated with laparoscopic rectal cancer surgery.

## 2. Methods

The present study enrolled 736 consecutive patients with rectal adenocarcinoma who underwent laparoscopic resection performed by a single surgeon (KHR) between August 2004 and February 2015. All included patients had histologically confirmed rectal adenocarcinoma and primary anastomosis. Conventionally, the rectum is divided into three parts based on the anatomic distance from the anal verge: the upper rectum (8–12 cm), mid rectum (4–8 cm), and lower rectum (0–4 cm). The exclusion criteria were the presence of a tumor location above 12 cm from the anal verge, anastomosis performed using a hand-sewn method, and the use of a diverting stoma. This study was reviewed and approved by the institutional review board of our hospital. The surgical technique of laparoscopic surgery for rectal cancer has been described previously [25]. Briefly, all patients first underwent mechanical bowel preparation. Five ports were used, and high ligation of the inferior mesenteric artery and vein was performed in most cases. The level of rectal transection was dependent on the location of the tumor. Total mesorectal excision was performed in most patients with tumors located below the peritoneal reflection. For upper rectal tumors, the rectum was transected 4-5 cm below the tumor. If there was uncertainty in the location of the lower margin of the tumor, a rigid sigmoidoscopy or digital rectal examination was used to determine the level of transection. The 60 mm bowel stapler was introduced through the 12 mm port in the right lower quadrant. If more than two loads were required to complete the distal transection, an additional 45 mm or 60 mm was used, which was done at the discretion of the surgeon. A double-stapling technique was applied in all patients, and rectal irrigation was performed with betadine solution.

AL was investigated at the surgeon's discretion on the basis of clinical symptoms of sepsis, including abdominal pain, tenderness, rebound tenderness, fever, and leukocytosis. It was suspected clinically if pus or fecal discharge was noted from the pelvic drain. All ALs were confirmed by using rigid sigmoidoscopy, abdominopelvic computed tomography, or operative findings.

### 2.1. Strategies to Reduce the Incidence of AL

Each of the strategies has been implemented since its initial use throughout the duration of the study period.

### 2.2. Application of Fibrin Glue

The application of fibrin glue over a stapled anastomosis site was routinely performed since August 2007. In this study, it was applied from the 155th consecutive patient. In the patients, 1-2 mL of Tissuecol (Baxter, Vienna, Austria) or Greenplast (Green Cross Corporation, Yongin, Korea) was used over the extraluminal anastomosis surface [14, 26].

### 2.3. Use of Reinforcing Sutures

Reinforcing sutures were used since January 2011. In this study, the sutures were used from the 397th consecutive patient. After anastomosis was performed, reinforcing 4-0 PDS (Ethicon Inc., Summerville, NJ) was used intraorally. At least two interrupted sutures were performed, and the sutures always included the point at which the circular and linear stapling line met.

### 2.4. Extended Medial-to-Lateral Splenic Flexure Mobilization (SFM)

Extended SFM was performed since December 2011. In this study, it was performed from the 480th consecutive patient. After ligation of the inferior mesenteric vein at the lower border of the pancreas, dissection was continued over the anterior surface of the pancreas to the splenic hilum until the lesser sac entered. Then, the splenic flexure was easily mobilized, the lateral ligament was divided, and the omentum was subsequently dissected from the colon [23].

### 2.5. Use of a Transanal Drainage Tube

A transanal drainage tube was used since January 2013. In this study, it was used from the 584th consecutive patient. Following anastomosis, a 10-Fr rubber catheter with two or three holes near the proximal tip was placed in the neorectum.

### 2.6. Statistical Analysis

The *χ*^2^ test was used to analyze categorical variables. A logistic regression model was used to identify the predictors of AL. Variables that were significant at *P* < 0.10 in the univariate analysis were considered in a backward stepwise multivariate logistic regression model. All statistical analyses were performed using R statistical software, version 3.1.3 (http://www.r-project.org/). Based on the multivariate logistic regression model, a nomogram was created using the rms package. The model performance for predicting AL was assessed by calculating the concordance index (C-index). A *P* value of <0.05 was considered significant in all the tests.

### 2.7. Calibration and Internal Validation of the Nomogram

The nomogram was validated internally with 240 bootstrap resamples. The validated function in the rms package was used to calculate the bias-corrected C-index, which was calculated by using Somers' Dxy rank correlation as follows: Dxy = 2 (C‐index − 0.5). Calibration of the nomogram for AL was performed by comparing the predicted ratio with the actual observed ratio of AL after bias correction.

### 2.8. RA-CUSUM

Because the estimated risk of AL varies significantly among patients, an adjustment for risk was performed. We used RA-CUSUM analysis on the basis of individual risk derived from the logistic regression model. The statistical principles were adapted from the tutorial by Steiner et al. [27] and our previous study [25]. CUSUM is calculated as follows: Sn = (*X*_i_ − p0_i_), where *X*_i_ = 0 for success (absence of AL) and 1 for failure (presence of AL), and p0_i_ denotes the predicted probability of failure for operation i. A multivariate logistic regression model was constructed, and the model-based probabilities (p0_i_) of AL in each individual patient were calculated for each combination of significant variables. The graph starts at zero and is plotted from left to right on the horizontal axis. The curve moves up by 1 − p0_i_ for every case with AL and down by p0_i_ for every case without AL. Thus, the RA-CUSUM chart is a very intuitive graphical representation of the surgical procedure.

## 3. Results

The present study included 736 patients. Among these patients, clinical AL occurred in 65 patients (8.8%) and relaparotomy was performed in 53 patients (7.2%). The detailed characteristics of the patients are presented in Table 1.

### 3.1. Development of Nomogram

On univariate analysis, male sex, a high American Society of Anesthesiologists (ASA) score, low rectal cancer, perioperative blood transfusion, and a long operation time were identified as significant risk factors for AL (Table 1). On multivariate analysis, male sex, a high ASA score, low rectal cancer, perioperative blood transfusion, and a long operation time remained significant risk factors for AL (Table 2).

**Figure 1:**
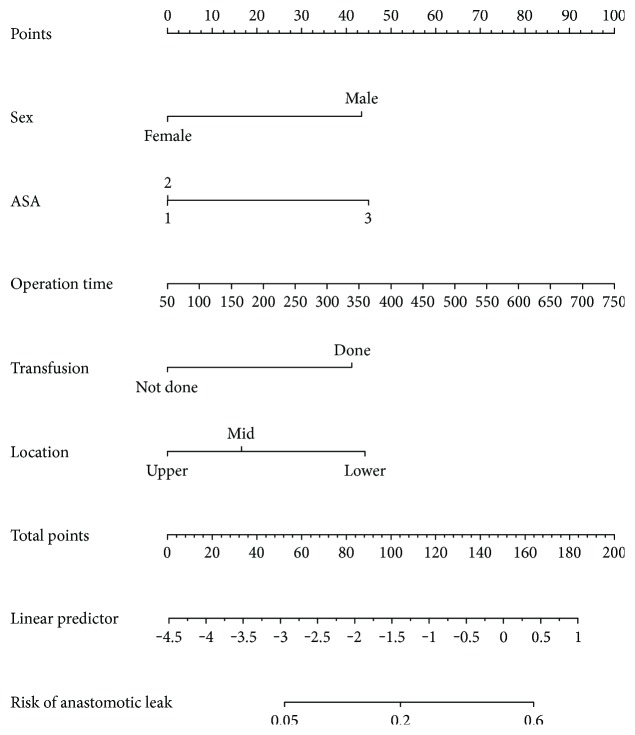
A nomogram for predicting postoperative anastomotic leakage after laparoscopic rectal cancer surgery. To use the nomogram, we first drew a vertical line to the top “Points” row to assign points for each variable. Then, we summed the total points and drew vertical line from the “Total points” row to obtain the probability of anastomotic leak.

A nomogram with significant risk factors was developed (Figure 1). The nomogram demonstrated that the operation time provided the greatest contribution to the occurrence of AL. The sum of each variable point was plotted on the total point axis, and we could draw a straight line to identify the predicted probability of AL. The C-index for this nomogram to predict AL was 0.753 (95% confidence interval, 0.690–0.816).

### 3.2. Calibration of the Nomogram

**Figure 2:**
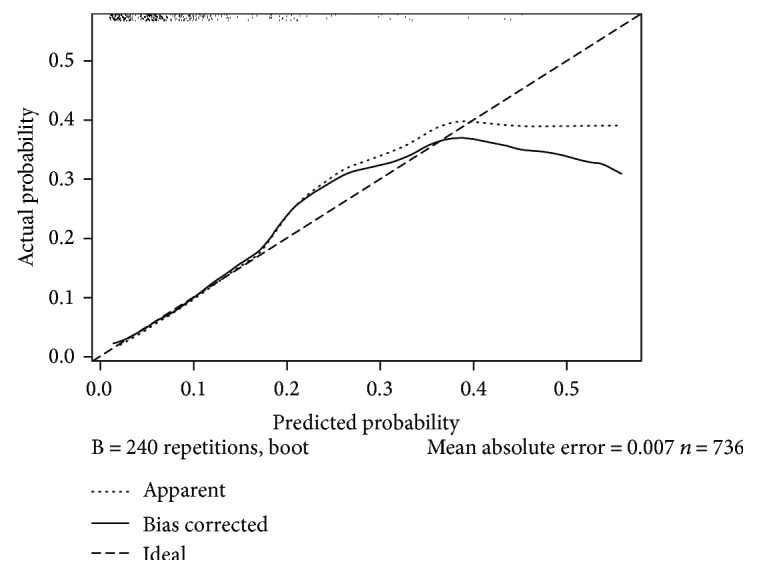
A calibration plot of the predicted and observed probabilities of anastomotic leakage after laparoscopic rectal cancer surgery. The *x*-axis indicates the predicted probability of anastomotic leakage, and the *y*-axis indicates the actual observed rate of anastomotic leakage.

The calibration plots showed that the model was very close to the ideal (Figure 2), especially for the relatively low risk group and that it had a bias-corrected C-index of 0.742.

### 3.3. Determination of an Effective Strategy for Reducing AL

**Figure 3:**
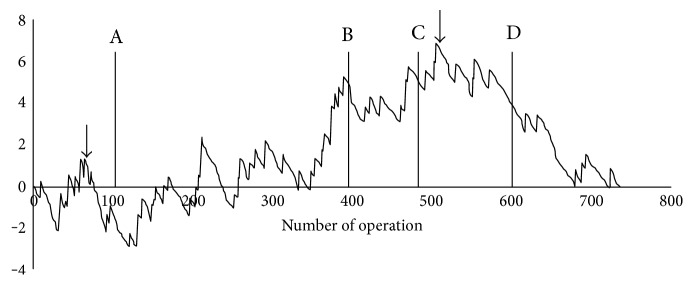
Risk-adjusted cumulative sum curve analysis for anastomotic leakage after laparoscopic rectal cancer surgery. The cut-off points were at the 70th case and the 500th case. Each of the strategies has been implemented since its initial use throughout the duration of the study period. A: application of fibrin glue, B: use of reinforcing sutures, C: extended medial-to-lateral splenic flexure mobilization, and D: use of a transanal drainage tube.

Based on the individual probability for AL after laparoscopic rectal cancer surgery, the RA-CUSUM graphical slope was determined and plotted according to the final surgical outcome. The RA-CUSUM graph for AL is presented in Figure 3. Marked cut-off points were identified at the 70th operation and 510th operation. On the basis of these cut-off points, we defined the first part as the learning curve for laparoscopic rectal cancer surgery and the second part as the protective role of extended SFM, which was performed from the 480th operation for AL. Extended SFM, which decreased anastomosis tension, and the surgeon's learning curve played important roles in the reduction of AL after laparoscopic rectal cancer surgery.

## 4. Discussion

We constructed a nomogram for predicting AL and found that extended SFM and the surgeon's learning curve played important roles in the reduction of AL after laparoscopic rectal cancer surgery. These findings may have clinical implications in the careful selection of candidates for diverting ileostomy based on our nomogram. Additionally, we suggest that extended SFM could be considered to reduce anastomosis tension when a more extended rectal resection is needed, for example, in patients with low rectal cancer and those preoperatively treated with CRT. To our knowledge, this is the first study that has used a nomogram and RA-CUSUM analysis to evaluate whether a surgical strategy can reduce AL after laparoscopic rectal cancer surgery.

The overall clinical AL rate in this study was 8.8%, and 81.5% of the patients underwent surgical intervention. The AL rate in this study is comparable to the rates of previous studies (3–21%) and the rates after laparoscopic surgery for rectal cancer [12–14, 18].

Our study found that male sex, a high ASA score (≥3), low rectal cancer, perioperative blood transfusion, and a long operation time were risk factors for AL. These risk factors can be categorized as patient-related factors (sex and ASA score), tumor-related factors (tumor location), and surgery-related factors (operation time and blood transfusion). The risk of AL was 3.7-fold higher in male patients than that in female patients in this study. This was also noted in previous studies [12, 15, 18, 28], and the higher risk in male patients might be associated with a more narrow pelvic space in male patients than in female patients. The ASA score has been reported to be associated with a high rate of wound-healing failure [15]. Bertelsen et al. [28] could not identify a significant association between the ASA score and AL; however, only patients with ASA scores of 1 and 2 were included in their study. Some authors have reported that an ASA score of at least 3 was independently related with a high risk of AL [29, 30]. In our study, as there was no significant difference between ASA 1 and 2, we classified ASA scores into two categories (≥3 and ≤2). A mildly debilitated physical status did not increase the risk of AL. Among the tumor features, tumor location was identified as a unique predictor of AL. Tumor stage and tumor size have been reported to be significant risk factor for AL after rectal cancer surgery. However, the association between these factors and AL remains unclear. There might be a consistent relationship between tumor distance from the anal verge and AL. Jannasch et al. [31] reported a significant association between tumor stage and AL (*P* < 0.001). Similarly, Warschkow et al. [16] identified tumor stage as a risk factor for AL. However, Bertelsen et al. [28] reported that there was no significant association between tumor stage and AL incidence, which is similar to our finding. In our study, long operation time and perioperative blood transfusion were also identified as significant risk factors for AL after laparoscopic rectal cancer surgery. These two factors may represent difficult operative circumstances that can adversely affect anastomosis integrity [14, 18]. In addition, these factors might be associated with the surgeon's laparoscopic experience. The cut-off points of the learning curve for laparoscopic rectal cancer surgery have been shown to be based on the mean operative time (range, 50–90 cases) [25, 32, 33]. In our previous study, we demonstrated that the mean operative time was 240 min after performing 90 cases [25]. Some investigators have insisted that operative time alone is insufficient as a surrogate marker for laparoscopic surgery [34]. Based on the findings of the multivariate analysis, we suggest that operation time was associated with not only the surgeon's learning curve but also surgical morbidity.

The combination of risk factors is very important, and the use of a nomogram is simple. For example, patients with the lowest risk were estimated to have an AL risk of only 1.6% after surgery, while patients with the highest risk were estimated to have an AL risk of 68.0%, implying that risk adjustment is the most critical step for sequential statistical analysis. For the RA-CUSUM curve constructed in this study, the following possible scores were plotted in the graphical presentation; if AL occurred, the score is 0.984 for patients with the lowest risk and 0.32 for patients with the highest risk after surgery. The rising curve was approximately three times steeper for patients with the lowest risk than for those with the highest risk. We demonstrated two change points at the 70th case and the 505th case. We speculate that the former might be attributed to the surgeon's learning curve and the latter might be attributed to the protective effect of extended SFM for AL. Previous studies have assessed whether fibrin glue application [14, 26], extended SFM [23], or a transanal drainage tube [24] can reduce the incidence of AL. However, none of these studies demonstrated a significant association between the strategy and AL. Therefore, in the present study, we used a highly sophisticated statistical method (RA-CUSUM) to assess the association. The most important advantage of RA-CUSUM analysis is that it can detect a small deviation during the surgical process. Therefore, it can produce a signal change and quickly provide information on which variable has an impact on the outcome.

To determine the strategy that is the most appropriate for the reduction of AL after laparoscopic rectal cancer surgery, many factors have to be controlled and an expert surgeon is required. In the present study, we adjusted many nonmodifiable variables (patient and tumor factors) known to be associated with AL after rectal cancer surgery, using RA-CUSUM analysis. The surgeon could control tension at the anastomosis site, vascular supply, stapler use, and diverting stoma creation. We believe that reduction of tension at the anastomosis site is the most critical factor, and this can be assessed in the operating room. In our study, after more than 500 cases of laparoscopic rectal cancer surgery, analyses to determine whether the use of a transanal drainage tube could reduce the AL rate were performed. We did not find a significant association, even using a propensity score analysis [24]. The surgeon was considered to have sufficient surgical experience at this point, and more than 90% of the stapling procedures were performed using single firing, and most vascular ligations were performed at the origin of the inferior mesenteric artery, as described in the methods. When extended surgical resection and high ligation are needed for advanced rectal cancer, we strongly recommend extended SFM as the routine procedure. This procedure has the advantage of sufficient resection of the irradiated bowel, which will increase the likelihood of a stable anastomosis.

The present study has several limitations. First, preoperative CRT has been shown to be a potential risk factor for AL [16–18]. However, some prospective trials have reported that preoperative CRT does not influence the AL rate [2, 3, 35]. Therefore, it remains controversial whether preoperative CRT is a risk factor for AL. In our study, we not only failed to find a significant association between preoperative CRT and AL but also excluded the majority of patients treated with preoperative CRT. Actually, we considered preoperative CRT as a risk factor for AL and used a diverting stoma in most of the patients who had undergone preoperative CRT. Of the 736 patients included in this study, only 53 patients (7.2%) had undergone preoperative CRT. As a result, the patients included in this study were subjectively considered as having a low risk for AL by the surgeon during anastomosis, and this might have caused selection bias. This might explain the relatively wide discrepancy of the calibration curve in the high-risk patient group. A preventive diverting stoma was made in patients with high-risk factors, which might reduce the actual occurrence of AL when compared to its predictive probability. Second, external validation was not performed. However, the factors identified in this study are well known and have been evaluated previously.

## 5. Conclusion

Our nomogram for predicting AL after laparoscopic rectal cancer surgery might be helpful to identify the individual risk of AL. Furthermore, extended SFM might be the most appropriate strategy for reducing AL in patients treated with laparoscopic cancer surgery.

## Figures and Tables

**Table 1:** Univariate analysis of risk factors for anastomotic leakage in patients treated with laparoscopic rectal cancer surgery without diverting stoma (*n* = 736).

Variables	Number of anastomotic leakage/total patients (%)	*P*
Sex		0.002
Female	12/272 (4.4)	
Male	53/464 (11.4)	
Age, yr		0.999
≥70	29/330 (8.8)	
<70	36/406 (8.9)	
BMI (kg/m^2^)		0.839
<25	46/507 (9.1)	
≥25	19/229 (8.3)	
ASA score		<0.001
1	14/280 (7.8)	
2	41/517 (7.9)	
3	10/39 (25.6)	
AJCC stage		0.163
0-II	33/439 (7.5)	
II/IV	32/297 (10.8)	
Maximum tumor size (cm)		0.896
<4	24/320 (7.5)	
≥4	29/416 (7.0)	
Location of tumor		0.005
Upper	25/447 (5.6)	
Mid	16/215 (7.4)	
Low	12/74 (16.2)	
Operative time (min)		0.003
<240	37/624 (5.9)	
≥240	16/112 (14.3)	
Transfusion		<0.001
No	41/674 (6.1)	
Yes	12/62 (19.4)	
Neoadjuvant chemoradiation		0.051
No	42/651 (6.5)	
Yes	11/85 (12.9)	
Number of linear stapler firing		0.061
<2	20/376 (5.3)	
≥2	33/360 (9.2)	

AJCC: American Joint Committee on Cancer; ASA: American Society of Anesthesiologists, BMI: body mass index.

**Table 2:** Multivariate analysis of risk factors associated with anastomotic leakage.

Variables	Relative risk	95% CI	*P*
Sex			
Male	1		
Female	0.272	0.129–0.526	<0.001
ASA score			
1/2	1		
3	3.818	1.587–8.622	0.002
Location of tumor			
Upper	1		
Mid	1.757	0.913–3.331	0.085
Low	3.721	1.761–7.635	<0.001
Operative time (min)	1.343	1.082–1.668	0.008
Transfusion			
No	1		
Yes	3.495	1.624–7.172	<0.001

ASA: American Society of Anesthesiologists; CI: confidence interval.
